# Inhibition of Breast Cancer Cell Invasion by Ras Suppressor-1 (RSU-1) Silencing Is Reversed by Growth Differentiation Factor-15 (GDF-15)

**DOI:** 10.3390/ijms20010163

**Published:** 2019-01-04

**Authors:** Vasiliki Gkretsi, Maria Louca, Andreas Stylianou, George Minadakis, George M. Spyrou, Triantafyllos Stylianopoulos

**Affiliations:** 1 Biomedical Sciences Program, Department of Life Sciences, School of Sciences, European University Cyprus, 1516 Nicosia, Cyprus; 2 Cancer Biophysics Laboratory, Department of Mechanical and Manufacturing Engineering, University of Cyprus, 1678 Nicosia, Cyprus; louca.maria@ucy.ac.cy (M.L.); stylianou.c.andreas.1@ucy.ac.cy (A.S.); 3 Bioinformatics Group, The Cyprus Institute of Neurology and Genetics, 2370 Nicosia, Cyprus; 4 The Cyprus School of Molecular Medicine, 2370 Nicosia, Cyprus; georges@cing.ac.cy (G.M.S.)

**Keywords:** actin cytoskeleton, ILK, PINCH-1, PARVA, ROCK-1, Fascin-1, invasion, bioinformatics, Rho-GTPases

## Abstract

Extracellular matrix (ECM)-related adhesion proteins are important in metastasis. Ras suppressor-1 (RSU-1), a suppressor of *Ras*-transformation, is localized to cell–ECM adhesions where it interacts with the Particularly Interesting New Cysteine-Histidine rich protein (PINCH-1), being connected to Integrin Linked Kinase (ILK) and alpha-parvin (PARVA), a direct actin-binding protein. *RSU-1* was also found upregulated in metastatic breast cancer (BC) samples and was recently demonstrated to have metastasis-promoting properties. In the present study, we transiently silenced *RSU-1* in BC cells, MCF-7 and MDA-MB-231. We found that *RSU-1* silencing leads to downregulation of Growth Differentiation Factor-15 (*GDF-15*), which has been associated with both actin cytoskeleton reorganization and metastasis. *RSU-1* silencing also reduced the mRNA expression of *PINCH-1* and cell division control protein-42 (*Cdc42*), while increasing that of *ILK* and *Rac* regardless of the presence of GDF-15. However, the downregulation of actin-modulating genes *PARVA*, *RhoA*, Rho associated kinase-1 (*ROCK-1*), and *Fascin-1* following *RSU-1* depletion was completely reversed by GDF-15 treatment in both cell lines. Moreover, complete rescue of the inhibitory effect of *RSU-1* silencing on cell invasion was achieved by GDF-15 treatment, which also correlated with matrix metalloproteinase-2 expression. Finally, using a graph clustering approach, we corroborated our findings. This is the first study providing evidence of a functional association between *RSU-1* and *GDF-15* with regard to cancer cell invasion.

## 1. Introduction

Despite significant advances in breast cancer (BC)-related research and treatment, the most difficult issue faced by BC patients still remains the possibility of metastasis, which actually accounts for most deaths in women due to cancer [1]. In fact, despite the development of novel therapeutic interventions in dealing with BC, there is still no way to halt metastasis once it has initiated. That by itself renders imperative any research efforts in the area. In that regard, it has been long postulated that the extracellular matrix (ECM) and ECM-related adhesion proteins are important players in metastasis, being greatly disrupted in tumors. This disruption allows, in turn, dissociation of cancer cells from the initial tumor [2,3,4,5], invasion through adjacent tissues, intravasation into blood vessels, and the establishment of a metastatic tumor in a favorable environment close to or far away from the primary site [6]. Importantly, reorganization of the actin cytoskeleton is also actively happening during all phases of metastasis enabling cancer cells to respond accordingly to external stimuli, undergo actin cytoskeleton remodeling, epithelial to mesenchymal transition (EMT), and migrate or invade through surrounding tissues [7,8].

Interestingly, the connection between cell–ECM and actin cytoskeleton has an inherent mechanical component which promotes cancer cell metastasis [9,10]. Excessive accumulation of ECM proteins within the tumor leads to tumor stiffening and greater mechanical compressive forces exerted on cancer cells, which result in enhanced cell invasion and metastasis [6,9,10,11,12,13,14,15,16,17].

Ras Suppressor-1 (RSU-1), was recently shown to be upregulated in increased stiffness conditions [18]. RSU-1 is a protein found at cell–ECM adhesion sites, where it is known to interact with Particularly Interesting New Cysteine-Histidine rich protein (PINCH-1) [19]. Through binding to PINCH-1, RSU-1 is indirectly connected to Integrin-Linked Kinase (ILK), and alpha-parvin (PARVA) with the latter providing a link to actin cytoskeleton as it directly binds to actin [20,21,22]. RSU-1 was first identified as a suppressor of *Ras*-dependent oncogenic transformation [23], with a proven growth suppressor activity [23,24,25,26], while a connection was recently made between RSU-1 and Rho-GTPases [26] that are downstream targets of *Ras* [27,28]. Interestingly, its expression was recently shown to be significantly elevated in metastatic colon cancer samples compared with healthy controls [29] in metastatic BC samples compared with in situ and normal adjacent tissue [30], as well as in highly invasive liver cancer cell lines [31]. RSU-1 expression was also correlated with poor prognosis for distant metastasis-free survival as well as remission-free survival [18]. Moreover, elimination of *RSU-1* from BC tumor spheroids [18] and hepatocellular carcinoma cells [31] significantly inhibited their in vitro invasive capacity, suggesting that RSU-1 is a metastasis-promoting protein. However, its mechanism of action is still vague.

Growth Differentiation Factor-15 (GDF-15) is another molecule connecting actin cytoskeleton reorganization, mechanical compression and cancer. It was discovered and cloned as a member of the Transforming Growth Factor β (TGF-β) superfamily and many names have been assigned to it including macrophage inhibitory cytokine-1 (MIC-1) [32], placental bone morphogenetic protein (PLAB) [33], Placental Transforming Growth Factor Beta (PTGFB) [34] and Non-Steroidal Anti-Inflammatory Drugs NSAID-activated gene-1, *NAG-1* [35]. It is activated upon mechanical compression [36], and its expression closely follows changes in the actin cytoskeleton and cell morphology [37]. Last but not least, GDF-15 levels have been found elevated in the serum of patients with metastatic BC, prostate, and colon cancer [38,39] while its role with regard to cell invasion is controversial indicating a possible cell-type-specific mechanism of action [40,41,42,43,44,45]. In the present study, we investigated, for the first time, the connection between RSU-1 and GDF-15 in BC cell with regard to their metastatic potential using in vitro experimental approaches. We found that *RSU-1* silencing downregulates *GDF-15*, and inhibits cancer cell invasion, a phenomenon which is completely reversed by treatment of cells with human recombinant GDF-15 (hrGDF-15). Moreover, we show that this rescue effect is accompanied by respective reversal of gene expression only in actin-related genes *PARVA*, *RhoA*, *ROCK-1*, and *Fascin-1*, and we corroborate our findings using graph clustering.

## 2. Results

### 2.1. RSU-1 Silencing in MCF-7 and MDA-MB-231 Cells Downregulates PINCH-1 and PARVA, and Upregulates ILK

**Figure 1:**
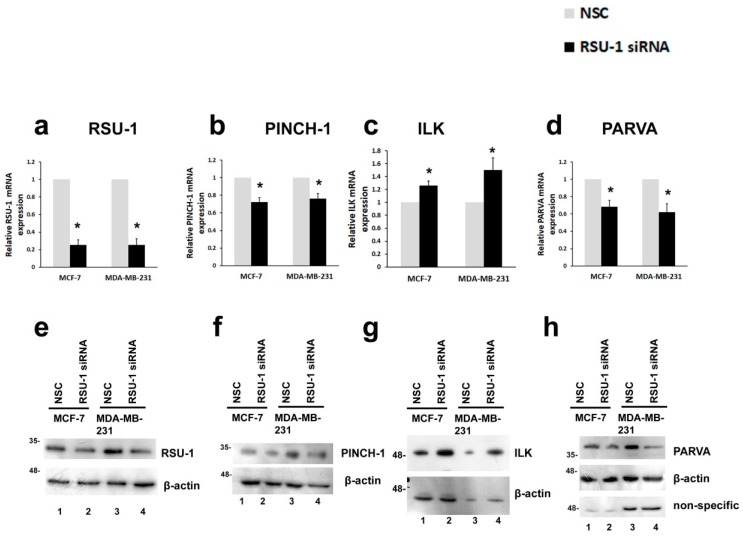
Ras suppressor-1 (*RSU-1*) silencing in MCF-7 and MDA-MB-231 cells downregulates Particularly Interesting New Cysteine-Histidine rich protein (*PINCH-1*) and alpha-parvin (*PARVA*) and upregulates Integrin Linked Kinase (*ILK*). (**a**–**d**) Relative mRNA expression in MCF-7 and MDA-MB-231 cells of *RSU-1* (**a**), *PINCH-1* (**b**), *ILK* (**c**), and *PARVA* (**d**) in cells transfected with non-specific control (NSC) or RSU-1 siRNA. Three independent real-time PCR experiments were performed, and data were analyzed using the ΔΔCt method and having the NSC-transfected cells as calibrators. Asterisks indicate statistically significant changes (*p*-value < 0.05). (**e**–**h**) Representative Western blots showing protein expression of *RSU-1* (**e**), *PINCH-1* (**f**), *ILK* (**g**), and *PARVA* (**h**) in MCF-7 and MDA-MB-231 cells. B-actin was utilized as loading control. Relative protein expression was quantified using the ImageJ software as described in the Materials and Methods section and Figure S1.

To investigate the role of *RSU-1* in BC cell metastasis, we used a siRNA-mediated silencing approach to inhibit the expression of *RSU-1* in two BC cell lines that differ in terms of their metastatic potential; the non-invasive MCF-7 cells and the highly invasive MDA-MB-231 cells. As shown in Figure 1, *RSU-1* was effectively silenced both at the mRNA (Figure 1a) and protein level (Figure 1e, compare lanes 1 and 2 and lanes 3 and 4 and Figure S1) as compared to the cells receiving the non-specific control siRNA sequence (NSC) that does not target any specific gene.

After successful silencing of the *RSU-1* gene, we set out to determine the expression of the *RSU-1*-associated genes, namely, *PINCH-1*, *ILK*, and *PARVA*. PINCH-1, a known direct interactor of RSU-1 was downregulated following *RSU-1* silencing at the mRNA level (Figure 1b) but did not seem to affect protein expression (Figure 1f and Figure S1b) while *PARVA*, which is also known to directly interact with actin cytoskeleton was downregulated both at the mRNA and protein level (Figure 1d,h and Figure S1d). However, *ILK* was found to be upregulated (Figure 1c,g and Figure S1c) upon *RSU-1* silencing, indicating that it is negatively regulated by *RSU-1*.

We also tested the expression of genes responsible for actin cytoskeleton stabilization as they are implicated in cancer cell migration and invasion, namely, the Rho-GTPases *Rac*, *RhoA*, *Cell division control protein-42* (*Cdc42*), *Rho-associated kinase-1* (*ROCK-1*), *Fascin-1* and *matrix metalloproteinase-2* (*MMP-2*). The Rho-family proteins Rac, Cdc42, and RhoA are small GTP-binding proteins that act as molecular switches, being activated by GTP binding and inactivated when bound to GDP according to signals received that normally include mechanical stress or cell–cell and cell–ECM adhesion, or growth factors and cytokines [46]. Thus, Rho-GTPases regulate cytoskeletal dynamics, motility, cytokinesis, cell growth, apoptosis, and transcriptional activity [46]. As shown in Figure S2, *Rac* mRNA expression was increased following *RSU-1* silencing (Figure S2a), whereas the mRNA expression of *RhoA* (Figure S2b), *Cdc42* (Figure S2c), *ROCK-1* (Figure S2d), and *Fascin-1* (Figure S2e) was significantly reduced in both cell lines. Finally, we tested the expression of *MMP-2* which is responsible for ECM degradation and is fundamental in cell invasion and found it to be also dramatically reduced following *RSU-1* silencing (Figure S2f).

### 2.2. RSU-1 Depletion from MCF-7 and MDA-MB-231 Cells Leads to Downregulation of GDF-15

**Figure 2:**
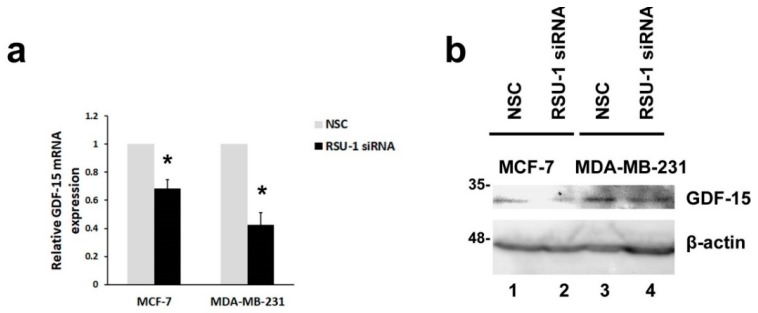
RSU-1 depletion from MCF-7 and MDA-MB-231 cells leads to downregulation of Growth Differentiation Factor-15 (GDF-15) at the mRNA and protein level. (**a**) Relative *GDF-15* mRNA expression in MCF-7 and MDA-MB-231 cells transfected with NSC or RSU-1 siRNA for at least 48 h. Six (6) independent real-time PCR experiments were performed, and data were analyzed using the ΔΔCt method and having NSC-treated cells as calibrators. Asterisks indicate statistically significant changes (*p*-value < 0.05). (**b**) Representative immunoblot showing GDF-15 protein expression in both BC cell lines treated with NSC or RSU-1 siRNA for at least 48 h. B-actin was utilized as loading control.

Since GDF-15 has been linked to actin cytoskeleton reorganization, and is activated by mechanical compression [36] and changes in the matrix and cell stiffness as well as cell morphology [37], we investigated whether silencing of *RSU-1* would have any effect on its expression. We found that *GDF-15* expression was significantly reduced following *RSU-1* silencing both at the mRNA (Figure 2a) and protein level (Figure 2b, compare lanes 2 and 4 with 1 and 3, and also Figure S2e) in both BC cell lines tested, suggesting a positive regulation by *RSU-1*.

### 2.3. Human Recombinant GDF-15 (hrGDF-15) Protein Reverses the Effect of RSU-1 Silencing on Gene Expression of Actin-Related Genes

**Figure 3:**
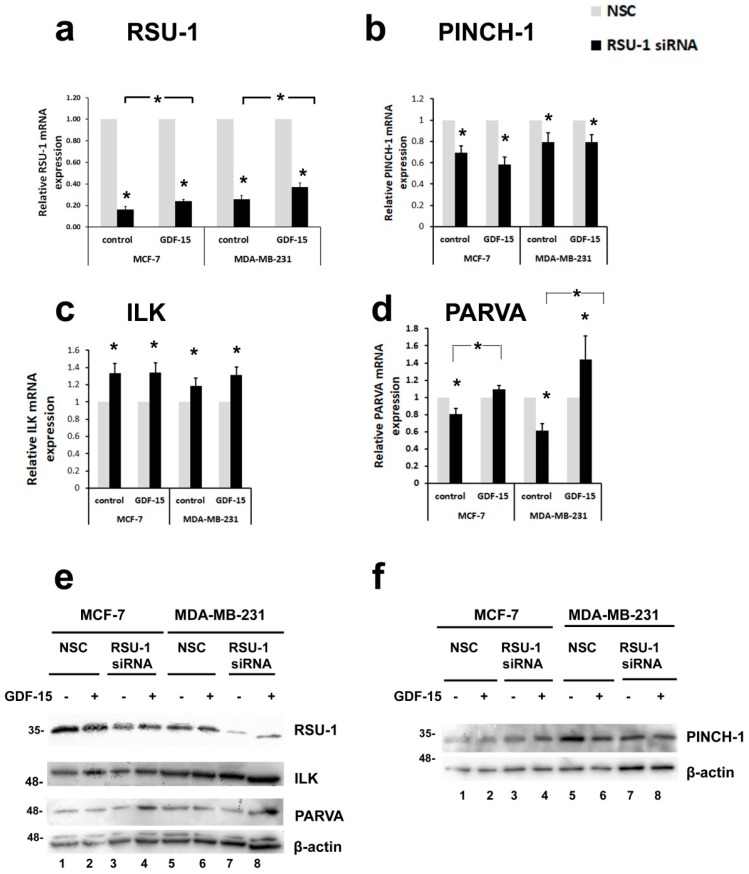
Treatment of breast cancer (BC) cells lacking RSU-1 with GDF-15 protein reverses the effect of RSU-1 silencing on the mRNA expression of actin-binding PARVA but not that of PINCH-1 and ILK. Relative mRNA expression of *RSU-1* (**a**), *PINCH-1* (**b**), *ILK* (**c**), and *PARVA* (**d**) in MCF-7 and MDA-MB-231 cells transfected with NSC or RSU-1 siRNA with or without 24 h treatment with human GDF-15 (10 ng/mL). Four (4) independent real-time PCR experiments were performed, and data were analyzed using the ΔΔCt method and having NSC-treated cells as calibrators. Asterisks denote statistically significant changes (*p*-value < 0.05). (**e**,**f**) Western blot showing the protein expression of RSU-1, ILK, and PARVA (**e**) and PINCH-1 (**f**) in MCF-7 and MDA-MB-231 cells transfected with NSC or RSU-1 siRNA with or without GDF-15. B-actin is used as loading control.

As RSU-1 is known to interact with PINCH-1 [19], it is not surprising that the components of the PINCH–ILK–PARVA complex are affected by *RSU-1* silencing. However, as the interplay between *RSU-1* and *GDF-15* is not currently defined and a connection between the two has not been made so far, we investigated their regulation further. To test whether they are interconnected, we examined if human recombinant GDF-15 (hrGDF-15) treatment would have the capacity to reverse the effect of *RSU-1* silencing on gene expression of BC cells. First, we treated BC cells with 10, 25, or 50 ng/mL GDF-15 for 24 h and found that no dose response effect was observed (Supplementary Figure S3). Thus, we proceeded with all subsequent experiments using 10 ng/mL GDF-15 treatment for 24 h. In that regard, we treated BC cells with RSU-1 siRNA or the control NSC, incubated them with the siRNA for 24 h and subjected them to GDF-15 or control treatment for an additional 24 h period. As seen in Figure 3a, *RSU-1* mRNA expression was reduced following silencing but GDF-15 treatment significantly elevated the *RSU-1* mRNA (Figure 3a) and protein (Supplementary Figure S4) level in both BC cell lines, indicating a cause and effect relationship between the two. This was also confirmed at the protein level as seen in Figure 3e (compare lanes 1, 2 to 3, 4 and 5, 6 to 7, 8 for RSU-1 silencing efficiency and lanes 1, 3, 5, 7 to 2, 4, 6, 8 for the effect of GDF-15 treatment, and Supplementary Figure S4 showing quantification of the immunoblots). *RSU-1* silencing also resulted in significantly reduced expression of its binding partner *PINCH-1* regardless of the presence or absence of GDF-15 treatment at the mRNA level (Figure 3b) but had no significant effect at the protein level (Figure 3f and Supplementary Figure S4). Similarly, *RSU-1* silencing led to significant upregulation of *ILK* at the mRNA level regardless of the presence or absence of GDF-15 treatment (Figure 3c) which was not true at the protein level (Figure 3e and Supplementary Figure S4). Interestingly, however, although *RSU-1* silencing in both cell lines significantly reduced the actin-binding *PARVA* mRNA expression (Figure 3d,e, compare lane 1 to 4 and lane 5 to 7 and Supplementary Figure S4), GDF-15 treatment reversed this effect in both cell lines (Figure 3d,e, compare lane 3 to 4 and lane 7 to lane 8 and Supplementary Figure S4).

**Figure 4:**
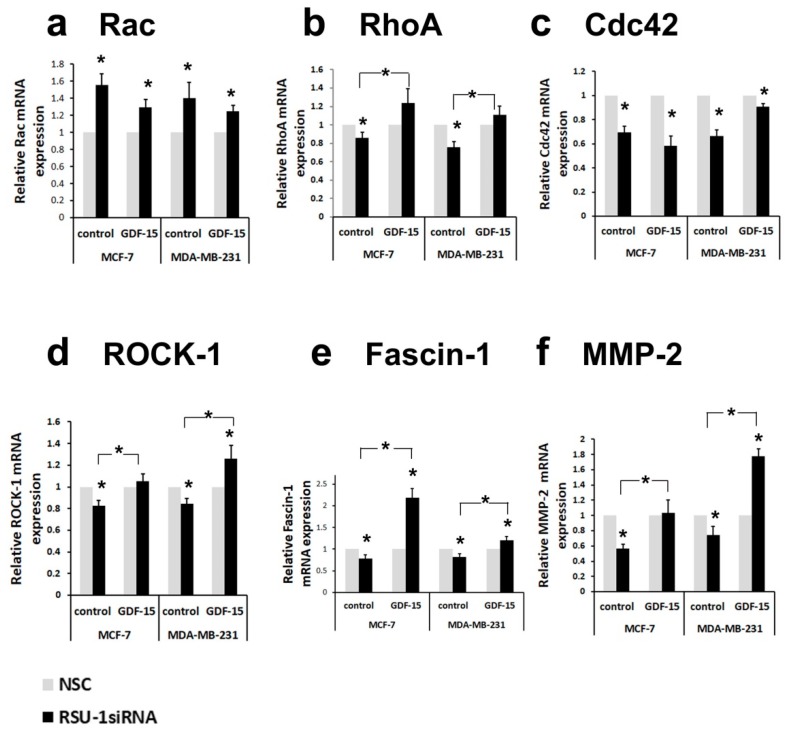
Treatment of BC cells lacking RSU-1 with human GDF-15 protein reverses the effect of RSU-1 silencing on the mRNA expression of actin-related genes *RhoA*, Rho associated kinase-1 (*ROCK-1*), and *Fascin-1*, as well as *MMP-2*. Relative mRNA expression of *Rac* (**a**), *RhoA* (**b**), *Cdc42* (**c**), *ROCK-1* (**d**), *Fascin-1*, (**e**) and *MMP-2* (**f**) in MCF-7 and MDA-MB-231 cells transfected with NSC or RSU-1 siRNA with or without 24 h treatment with human GDF-15 (10 ng/mL). Four (4) independent real-time PCR experiments were performed, and data were analyzed using the ΔΔCt method and having NSC-treated cells as calibrators. Asterisks denote statistically significant changes (*p*-value < 0.05).

Intrigued by this finding, we wondered whether other actin-related genes were similarly affected. The mRNA expression of *Rac* and *Cdc42* Rho-GTPases was increased (Figure 4a) and reduced (Figure 4c), respectively, regardless of the presence of GDF-15. Notably, however, the mRNA expression of genes that are directly related to actin cytoskeleton stabilization and migration/invasion was completely reversed by GDF-15 treatment. Thus, the inhibition in mRNA expression of *RhoA* (Figure 4b), *ROCK-1* (Figure 4d), *Fascin-1* (Figure 4e), was completely reversed by GDF-15 treatment in both cell lines, indicating a GDF-15-dependent mechanism of RSU-1 action on these molecules. Moreover, the same effect was observed in the expression of *MMP-2* (Figure 4f), known to be critically involved in cancer cell invasion.

### 2.4. Treatment of BC Cells Lacking RSU-1 with GDF-15 Rescues the Inhibitory Effect of RSU-1 Silencing on Cell Invasion

**Figure 5:**
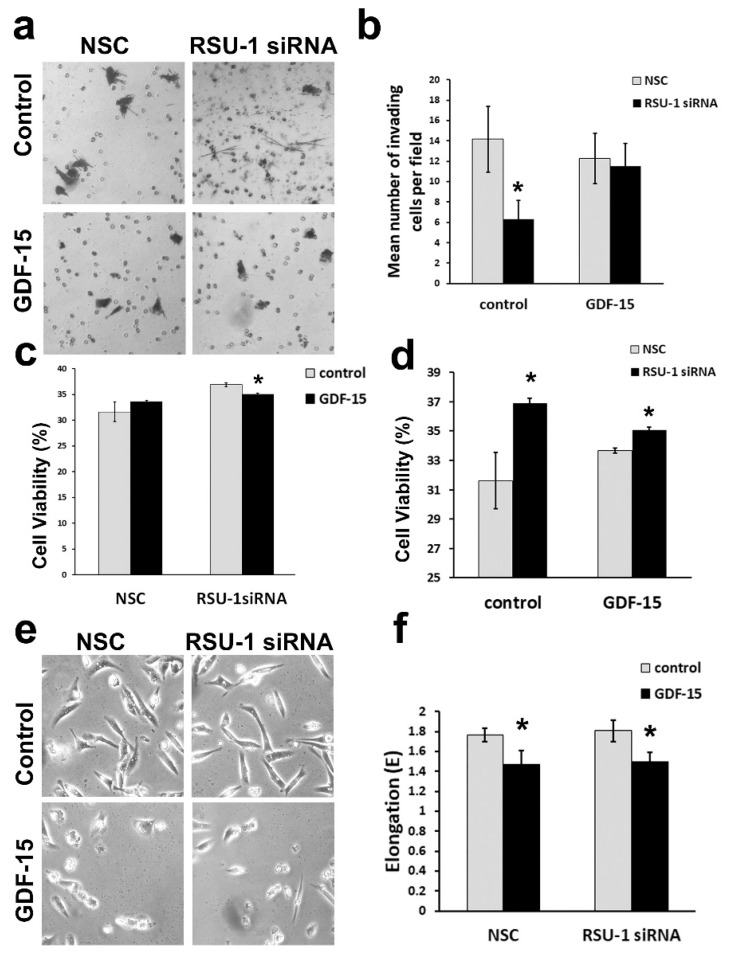
Treatment of BC cells lacking RSU-1 with human GDF-15 protein rescues the inhibition of cell invasion observed upon RSU-1 silencing. (**a**) Transwell invasion in MDA-MB-231 cells transfected with NSC or RSU-1 siRNA with or without human GDF-15 treatment (10 ng/mL). (**b**) Diagrammatic representation of the mean number of invading cells per field. Asterisks denote statistically significant changes (*p*-value < 0.05). (**c**,**d**) Cell viability of MDA-MB-231 cells following RSU-1 siRNA in the presence or absence of GDF-15 measured by Alamar blue assay as far as GDF-15 treatment is concerned (**c**) and as far as response to *RSU-1* silencing (**d**). (**e**) Cell morphology of MDA-MB-231 cells transfected with NSC or RSU-1 siRNA with or without human GDF-15 treatment as seen under the optical microscope (10× objective). (**f**) Diagrammatic representation of the mean cell elongation as measured by the elongation factor E. All asterisks indicate statistically significant changes (*p*-value < 0.05).

Based on our findings that GDF-15 treatment reverses the mRNA expression changes observed upon *RSU-1* silencing in actin cytoskeleton-related genes, we investigated whether GDF-15 treatment could also reverse the already known inhibitory effect of *RSU-1* silencing on cell invasion [18,31]. Thus, a set of transwell invasion experiments was performed only in the highly metastatic MDA-MB-231 cells lacking *RSU-1* in the presence or absence of GDF-15, as MCF-7 are non-invasive. As shown in Figure 5a,b, MDA-MB-231 cells lacking *RSU-1* exhibited considerably less invasion compared with the NSC, while treatment with GDF-15 completely rescued the invasive phenotype, proving that the effect of RSU-1 on cell invasion is GDF-15-dependent.

Next, to test whether the inhibitory effect seen in cell invasion was related to compromised proliferation, we performed cell viability assay using Alamar blue reagent. As shown in Figure 5c, GDF-15 treatment does not affect cell viability in NSC-treated cells and has a slightly inhibitory effect on *RSU-1*-siRNA-treated cells. However, when cell viability was assessed with regard to *RSU-1* silencing, it was verified that *RSU-1* silencing promotes cell proliferation (Figure 5d) in control cells. Interestingly, in GDF-15-treated cells, cell viability was still increased although not as dramatically (Figure 5d) most likely due to the reduction exerted by GDF-15 treatment seen in Figure 5c.

Moreover, cell morphology was examined in the same cells and as seen in Figure 5e,f although *RSU-1* silencing did not affect cell morphology, GDF-15 treatment affected cell shape by reducing cell elongation regardless of the presence or absence of RSU-1.

### 2.5. Graph Clustering Confirms a Connection between Ras Signaling, BC, Cell–ECM Adhesions, and Actin Cytoskeleton

**Figure 6:**
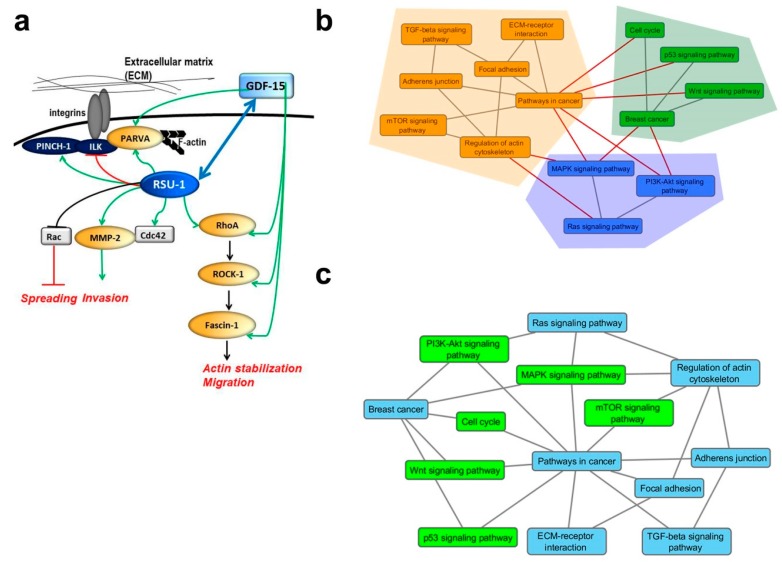
Proposed model of molecular interactions and clustering analysis. (**a**) Diagrammatic overview of the interactions implicating RSU-1 in basic metastasis-related cellular properties based on our findings. All arrows and lines in black designate known connections based on the literature, while green and red arrows and lines represent connections proposed based on the findings of the present work. RSU-1 has been previously shown to inhibit cell proliferation [23,24,30], and spreading through inhibition of Rac [47] and promote cell invasion [18,31], while data on cell migration seem to be dependent on the cell type or system used [47,48]. Data from the present work suggest that RSU-1 positively regulates PINCH-1 and PARVA whereas it negatively regulates ILK while it also seems to activate GDF-15 providing another link towards cell invasion. The blue bi-directional arrow connecting RSU-1 and GDF-15 conveys the main finding of this study, that RSU-1 depletion inhibits GDF-15 (Figure 2), but GDF-15 treatment also upregulates RSU-1 (Figure 3). (**b**) Clusters of pathways each one shaded by a different color. Herein the edge betweeness community detection algorithm detects communities by progressively removing edges from the original network that are most likely “between” communities. The black edges (lines) refer to the connections between nodes that form the identified clusters, while the red ones refer to those that have been excluded by the algorithm. (**c**) Complementary network of pathways: the nodes refer to the pathway names while the edges refer to the functional connection between them as provided by the Kyoto Encyclopedia of Genes and Genomes (KEGG) reference network. The blue nodes refer to the initial pathways identified in this work while green nodes refer to the complementary pathways obtained by the shortest path algorithm described in the text.

Based on the findings of the present study, we propose a mode of interaction between *RSU-1*, *PINCH-1*, *ILK*, *PARVA*, *GDF*-15, *Rho-GTPases*, *ROCK-1*, and *Fascin-1* with regard to basic cellular properties defining the metastatic identity of a cell, summarized in Figure 6a. According to this, RSU-1 stabilizes actin cytoskeleton and promotes cell invasion through the RhoA-ROCK-1-Fascin-1 pathway, which is also positively regulated by GDF-15 in BC cells. Therefore, when cells lack *RSU-1*, they have impaired motility and invasion, but this phenomenon is reversible upon addition of GDF-15.

To further enhance our understanding of the molecular interactions under study, we used a bioinformatics approach based on graph theory to reveal the direct connections between pathways of interest and highlight communities (clusters) of pathways [49,50] utilizing the edge betweeness clustering algorithm [51]. Bioinformatics analysis confirmed the general idea of our proposed model (Figure 6b). Pathways in cancer were identified according to Kyoto Encyclopedia of Genes and Genomes (KEGG) repository to be highly connected to pathways related to cell–cell adhesion (adherens junctions), cell–ECM adhesion (focal adhesion), ECM–receptor interaction and regulation of the actin cytoskeleton (pink cluster) while also being connected to *Wnt* and *p53*-related pathways (blue cluster) as well as the major pro-survival pathways of mitogen-activated protein kinase (*MAPK*), *Akt* and *Ras* (green cluster). Based on our findings *RSU-1* connects the *Ras* signaling pathway with actin cytoskeleton regulation potentially through *GDF-15* (Figure 6b,c).

## 3. Discussion

RSU-1, a known suppressor of *Ras*-transformation with growth suppressor properties [23,24,25,26], is also found to be localized to cell–ECM adhesions where it interacts with PINCH-1 [19], being connected to the PINCH–ILK–PARVA complex and, thus, to the actin cytoskeleton [20,21,22]. Recently, however, RSU-1 has been gaining ground in the field of cancer metastasis [18,52,53]. Moreover, it was demonstrated to have metastasis-promoting properties as its depletion from BC [18] and hepatocellular carcinoma [31] cells significantly impaired their in vitro invasive capacity. However, little is known with regard to the implicated molecular mechanisms. In the present study, we investigated the effect of *RSU-1* silencing on the expression of its binding partners, the PINCH–ILK–PARVA complex as well as several basic actin cytoskeleton regulators. We found that, as a response to *RSU-1* silencing in both BC cell lines, PINCH-1 was downregulated at the mRNA level but was not affected at the protein level while PARVA was downregulated both at the mRNA and protein level (Figure 1c,d,g,h, respectively and Supplementary Figure S1). ILK, on the other hand, was found to be elevated (Figure 1e,f and Supplementary Figure S1). While upregulation of PINCH-1 in squamous cell carcinoma has been considered to predict metastasis [54] and is associated with poor prognosis in laryngeal carcinoma patients [55], no study has demonstrated its direct effect on cell invasion. ILK is more established in the field and has been proposed as a potential anti-cancer therapeutic target [56] being involved in tumor growth and invasion [57,58] in many types of cancers while there was also a report of a suppressive effect seen on BC cells following ectopic expression of ILK [59]. As far as PARVA is concerned, it has been shown to promote cell invasion [60] and lymph node metastasis [61]. Moreover, RhoA, ROCK-1, and Fascin-1, which are critical regulators of the actin cytoskeleton, have been long connected with an invasive phenotype [62,63,64] and are elevated in human BC samples [64]. In the present work, we show that *RSU-1* exerts a positive type of regulation on PINCH-1 and PARVA (Figure 6a). Moreover, we demonstrate that *RSU-1* silencing enhances the expression of *Rac* (Figure S2a), while at the same time exhibits an inhibitory effect on the expression of *RhoA* (Figure S2b), *Cdc42* (Figure S2c), *ROCK-1* (Figure S2d), and *Fascin-1* (Figure S2e). These findings are certainly justified as the *RhoGTPases* have different effectors through which they exert their action on cells [46]. Moreover, we also showed, for the first time, that *RSU-1* silencing leads to significant downregulation of *GDF-15*, in both cell lines (Figure 2). GDF-15 has also been found to mediate cell invasion in BC cells [65]. All in all, the genes whose expression is reduced following *RSU-1* silencing (namely PARVA, GDF-15, RhoA, ROCK-1, and Fascin-1, MMP-2) have a common profile; they are all related to actin cytoskeleton organization, and they have all been previously associated with the promotion of cell invasion. This clearly indicates that RSU-1 exerts its invasion-promoting actions through activation of these genes (Figure 6a).

Importantly, being that *GDF-15* has been associated with actin cytoskeleton reorganization [36], changes in matrix stiffness and cell morphology [37], as well as cancer progression [38,42,66,67], its regulation by *RSU-1* seemed of particular importance. Hence, to determine whether *RSU-1* exerts its actions on invasion through GDF-15, we eliminated *RSU-1* from BC cells by siRNA and the following day treated them with GDF-15 protein for an additional 24 h. Our results showed that GDF-15 treatment significantly elevated *RSU-1* mRNA levels in both BC cell lines, giving the first hint of a cause and effect relationship between the two (Figure 3a). Thus, *RSU-1* silencing reduced the mRNA expression of *PINCH-1* (Figure 3b) and *Cdc42* (Figure 4c), and increased that of *ILK* (Figure 3c) and *Rac* (Figure 4a) regardless of the presence or absence of GDF-15 treatment. Notably, though, *RSU-1* depletion downregulated *PARVA* (Figure 3d), *RhoA* (Figure 4b), *ROCK-1* (Figure 4d), and *Fascin-1* (Figure 4e) but GDF-15 treatment was able to completely reverse this downregulation. Interestingly, this effect was confirmed at the protein level as well for *PARVA* (Figure 3e). This is of particular interest, as from the genes tested, only the ones that had a direct connection to the actin cytoskeleton (e.g., *PARVA*) or had the capacity to modulate it (e.g., *RhoA*, *ROCK-1*, *Fascin-1*) were responsive to GDF-15 treatment after *RSU-1* silencing. More specifically, *Cdc42* has been connected to transcriptional activation and DNA synthesis [46], while it is not involved in the formation of actin-rich structures responsible for migration such as the filopodia and lamellipodia, nor it is important for migration overall [68]. *Rac* on the other hand is fundamental in cell survival and spreading [46] and our results (Figure S2a) verify a previous study showing that depletion of *RSU-1* increases *Rac* expression [47]. *RhoA*, on the other hand, is the *RhoGTPase* that is fundamentally involved in actin cytoskeleton remodeling, cell migration, and invasion [46], thus, it is not surprising that *RhoA* and its downstream effectors *ROCK-1* and *Fascin-1* are positively regulated by *RSU-1*.

Furthermore, our data showed that GDF-15 treatment also reverses the reduction in *MMP-2* mRNA expression caused by *RSU-1* elimination (Figure 4f). Interestingly, GDF-15 treatment affected cell shape regardless of the presence or absence of *RSU-1* by promoting a more spherical shape compared to the characteristic elongated cell shape of MDA-MB-231 cells, indicating that GDF-15 indeed interferes with actin cytoskeleton reorganization affecting basic cellular functions. Moreover, GDF-15 does not seem to significantly affect cell viability, as it was promoted in cells lacking RSU-1 regardless of the presence or absence of GDF-15 (Figure 5c). In that regard, the fact that GDF-15 treatment completely reversed the inhibitory effect of *RSU-1* silencing on cell invasion of MDA-MB-231 cells (Figure 5) firmly suggests that RSU-1 and GDF-15 are also functionally coupled both contributing to cell invasion.

Although *GDF-15* is known to promote cytoskeletal rearrangements leading to tumor dissemination [66], to promote metastasis of colon cancer cells [42] and prostate cancer cells through *RhoA* [69] and to be increased in association with morphology changes during metastasis [37], this is the first study providing evidence of functional association between *RSU-1* and *GDF-15* with regard to BC cell invasion.

Based on our results, we propose a mode of interaction between *RSU-1*, *PINCH-1*, *ILK*, *PARVA*, *GDF*-15 and *Rac*, *RhoA*, *ROCK*, and *Fascin-1* with regard to basic metastasis-regulating processes, summarized in Figure 6a. Our data support the idea that RSU-1 activates actin stabilization and, thus, invasion through the RhoA-ROCK-1-Fascin-1 pathway, which is positively regulated by GDF-15 in BC cells. Thus, depletion of *RSU-1* leads to decreased motility and invasion, which is completely reversed by GDF-15.

Finally, using a graph clustering approach, we corroborated our findings and line of investigation demonstrating that pathways in cancer identified in KEGG repository are highly connected to pathways related to cell–cell adhesion (adherens junctions), cell–ECM adhesion (focal adhesion), ECM–receptor interaction and regulation of the actin cytoskeleton (Figure 6b, pink cluster) while also being connected to the major pro-survival pathways of *MAPK*, *Akt*, and *Ras* (Figure 6b, green cluster). *RSU-1*, based on our data, most likely serves as the connector between the *Ras* signaling pathway and actin cytoskeleton regulation (Figure 6b,c).

The present analysis provides new insights into the molecular mechanism underlying the role of RSU-1 in BC cell metastasis, highlighting the involvement of Akt, MAPK, and mTOR signaling pathways in BC. Of course, a broad field of investigation is waiting to be explored with many more questions that need to be answered. For instance, apart from the pathways highlighted in Figure 6, it would be interesting to determine whether the newly-identified receptor of GDF-15, namely GDNF-family receptor a-like (GFRAL) [70,71,72,73], mediates the GDF-15-induced rescue of the inhibitory effect of *RSU-1* depletion on BC cell metastasis. Another aspect that also needs to be investigated is the effect on PINCH-1 as it seems to be regulated by RSU-1 at the transcriptional but not the translational level which obviously highlights the complexity of the interaction involved. The current work provides a novel mechanism of BC cell invasion that was verified in vitro and, for the first time, connects RSU-1 with GDF-15 in the context of BC metastasis. However, further studies using in vivo animal models are necessary to prove the relevance of the proposed mechanism in the whole animal. Finally, investigation of the proposed molecular pathway in other types of cancer cells would also give information on how universal this connection really is.

## 4. Materials and Methods

### 4.1. Antibodies and Reagents

Anti-ILK, anti-PINCH-1, anti-PARVA and anti-GDF-15 antibodies were purchased from Cell Signaling Technology (Danvers, MA, USA) and anti-β-actin antibody from Sigma-Aldrich (St. Louis, MO, USA). Anti-RSU-1 rabbit polyclonal antibody was kindly provided by Dr. Mary Lou Cutler, Professor at the Uniformed Services University of the Health Sciences (Bethesda, MD, USA). GDF-15 human recombinant protein (hrGDF-15) was purchased from R&D systems (Minneapolis, MI, USA), SuperSignal West Fempto chemiluminescent reagent was purchased from Pierce (Rockford, IL, USA) and Mitomycin C was obtained from Sigma-Aldrich (St. Louis, MO, USA).

### 4.2. BC Cell Lines

BC cell lines MCF-7 and MDA-MB-231 were purchased from ATCC (Manassas, VA, USA). All cells were grown in Dulbecco’s Modified Eagle Medium supplemented with 10% Fetal Bovine Serum (FBS), 1% Glutamine and 1% Penicillin/Streptomycin, and incubated in a CO_2_-incubator at 37 °C.

### 4.3. siRNA Transfection

BC cells were transfected with 100 nM non-specific control (NSC) siRNA or siRNA against RSU-1 (Santa Cruz Biotechnology, Dallas, TX, USA) using the Lipofectamine 2000 reagent (Invitrogen Life Technologies, Carlsbad, CA, USA). Transfected cells were harvested 48 h following transfection while knockdown efficiency was assessed by immunoblotting and real-time PCR as specified in each experiment.

### 4.4. GDF-15 Stimulation in siRNA-Transfected Cells

MCF-7 and MDA-MB-231 cells were grown until they reached 80% confluence and were subjected to siRNA-mediated transfection, as described above, in low serum-containing medium (DMEM supplemented with 0.5% FBS). After a 24 h incubation period, human hrGDF-15 (10 ng/mL) or the same amount of the respective control, was added. The solvent in which GDF-15 was dissolved (4 mM HCI containing 0.1% bovine serum albumin) was used as the control. Cells were cultured under these conditions for an additional 24 h period and then processed for further assays.

### 4.5. RNA Isolation and Real-Time PCR

Total RNA extraction from BC cells, RNA purification, cDNA synthesis and gene expression analyses at the mRNA level were performed as described previously [18]. All primers used are shown in Table 1. Relative gene expression at the mRNA level was finally quantified using the ΔΔCt method where cells transfected with NSC served as calibrators.

### 4.6. Protein Extraction and Western Blot Analysis and Quantification

For protein expression analysis, a standard immunoblotting protocol was followed as described previously [18]. Protein expression was normalized to β-actin using the National Institute of Health (NIH) ImageJ software. At least two immunoblots from two independent experiments were used for each quantification, as indicated in the respective figure legends. A *p* value of 0.05 was considered as a statistically significant difference when comparing the band intensity in various immunoblots.

### 4.7. Cell Invasion Assays

Transwell invasion assay [74] was analyzed using the BD biosciences (San Jose, CA, USA) tumor invasion system with matrigel coated inserts. Briefly, cells were pre-treated with 0.5 mg/mL mitomycin C for 2 h before the experiment. Subsequently, they were trypsinized and suspended in DMEM containing 1 mg/mL BSA at a concentration of 5 × 10^5^ cells/mL, while complete serum-containing medium was placed at the bottom chamber. Cells were then incubated at 37 °C for 18 h, and non-invading cells that remained on the upside of the filter were removed. The invading cells were fixed with paraformaldehyde, stained with 0.1% crystal violet for 30 min and photographed under an optical microscope (Nikon Eclipse, Amsterdam, Netherlands) equipped with a digital camera. Pictures were taken from five (5) randomly selected optical fields under the 10× objective. The mean number of invading cells was quantified by counting cells from the five microscopic fields. Five (5) independent experiments were performed. Asterisks denote statistically significant changes (*p* < 0.05).

### 4.8. Cell Viability

MDA-MB-231 cell viability following RSU-1 siRNA with or without hrGDF-15 treatment were subjected to Alamar blue assay (Thermo Fisher Scientific, Waltham, MA, USA) [75] 24 h post-siRNA treatment according to the company’s guidelines. Absorbance was measured using a Rayto microplate spectrophotometer, and three (3) independent experiments were performed.

### 4.9. Cell Elongation

A Nikon Eclipse TS100 inverted microscope (equipped with an Olympus XC50 Color CCD camera and a Nikon Ph1 DL 10× 0.25 phase microscope objective lens) was used to assess cell elongation from live cells, as described previously [76]. Briefly, pictures of individual cells treated with NSC or RSU-1 siRNA with or without hrGDF-15, were analyzed with NIH ImageJ software and *elongation factor E* was measured [76,77] as a means to describe the extent to which the equimomental ellipse is lengthened or stretched out [78,79].

### 4.10. Graph Theory Analysis

For the detection of disease-related clusters of molecular mechanisms, we used a recently introduced methodology utilizing a reference pathway network [80,81,82]. The specific reference network is based on the functional connection among pathways that can be found in the Kyoto Encyclopedia of Genes and Genomes (KEGG) repository (Available online: http://www.genome.jp/kegg/, last accessed in March 2018), which is a well-established database for obtaining pathway information. Specifically, for a given set of pathways, this methodology identifies and adds the key pathways (nodes) that ensure the minimal connectivity of the network. For this, a specific algorithm calculates all the shortest paths within the reference network that interconnect these nodes together [80,81,82]. This process results in an extended network of pathways able to provide enriched functional information related to the phenotype under study. When working on a given set of genes, enrichment analysis is performed using the well-known *EnrichR* package [49,50], to obtain the list of initial pathways and then proceed with the creation of connected pathway networks. Furthermore, the specific methodology also provides the option of grouping the pathways into clusters through a series of community-detection algorithms. In this work, we employed the edge betweeness clustering algorithm [51].

### 4.11. Statistical Analysis

A *t* test was used for comparison of means between two groups (e.g., NSC versus RSU-1 siRNA-treated cells) in all experiments performed. A *p*-value < 0.05 was considered statistically significant.

## 5. Conclusions

RSU-1 is a cell–ECM adhesion protein that has been recently linked to BC metastasis being upregulated in BC samples [30] while its depletion severely impairs cancer cell invasion in vitro [18,31]. GDF-15 has also been found to be upregulated in many cancer types, and it is even detected in the plasma of cancer patients [38,39,41,69,83]. Furthemore, it has been associated with changes in the actin cytoskeleton. However, a connection between the two has not been made so far. In the present work, we show that elimination of *RSU-1* from two BC cell lines impairs cancer cell invasion which is completely reversed by GDF-15 treatment. Last, but not least, gene expression of actin regulating genes *PARVA*, *RhoA*, *ROCK-1*, and *Fascin-1* as well as *MMP-2* but not other *Rho-GTPases* or RSU-1 interacting genes followed the same pattern of expression being downregulated by *RSU-1* silencing and recovered their expression after GDF-15 treatment, suggesting that they are likely involved in the same molecular pathway, as was also demonstrated by a graph clustering approach. This is the first study providing evidence of a functional association between *RSU-1* and *GDF-15* with regard to BC cell invasion and in relation to actin–cytoskeleton-related genes providing new insights into the mechanisms of BC cell metastasis.

## Figures and Tables

**Table 1:** Nucleotide sequence of the primers used for mRNA expression analysis.

Primer Name	Sequence
**Cdc42**	Forward: 5′-GCCCGTGACCTGAAGGCTGTCA-3′
	Reverse: 5′-TGCTTTTAGTATGATGCCGACACCA-3′
**Fascin-1**	Forward: 5′-AGCTGCTACTTTGACATCGA-3′
	Reverse: 5′-TCATGAGGAAGAGCTCTGAGT-3′
**GDF-15**	Forward: 5′-TCAAGGTCGTGGGACGTGACA-3′
	Reverse: 5′-GCCGTGCGGACGAAGATTCT-3′
**ILK**	Forward: 5′-GACATGACTGCCCGAATTAG-3′
	Reverse: 5′-CTGAGCGTCTGTTTGTGTCT-3′
**MMP-2**	Forward: 5′-ATGACAGCTGCACCACTGAG-3′
	Reverse: 5′-AGTTCCCACCAACAGTGGAC-3′
**PARVA**	Forward: 5′-CAATTCGACTCCCAGACCAT-3′
	Reverse: 5′-TGGTCGAACAAGGTGTCAAA-3′
**PINCH-1**	Forward: 5′-CCGCTGAGAAGATCGTGAAC-3′
	Reverse: 5′-GGGCAAAGAGCATCTGAAAG-3′
**Rac1**	Forward: 5′-AACCAATGCATTTCCTGGAG-3′
	Reverse: 5′-CAGATTCACCGGTTTTCCAT-3′
**RhoA**	Forward: 5′-CGGGAGCTAGCCAAGATGAAG-3′
	Reverse: 5′-CCTTGCAGAGCAGCTCTCGTA-3′
**ROCK-1**	Forward: 5′-ACCTGTAACCCAAGGAGATGTG-3′
	Reverse: 5′-CACAATTGGCAGGAAAGTGG-3′
**RSU-1**	Forward: 5′-AGGCCACAGAGCAAGGTCTA-3′
	Reverse: 5′-CGTGCAATCTCAAAAGCTCA-3′
**β-actin**	Forward: 5′-CGAGCACAGAGCCTCGCCTTTGCC-3′
	Reverse: 5′-TGTCGACGACGAGCGCGGCGATAT-3′

## Supplementary Materials

Supplementary materials can be found at http://www.mdpi.com/1422-0067/20/1/163/s1.

## Abbreviations

BC	Breast Cancer
Cdc42	cell division control protein-42
ECM	extracellular matrix
EMT	epithelial to mesenchymal transition
GDF-15	Growth Differentiation Factor-15
GFRAL	GDNF-family receptor a-like
ILK	integrin-linked kinase
KEGG	Kyoto Encyclopedia of Genes and Genomes
MAPK	mitogen-activated protein kinase
MMP	Metalloproteinases
NSC	non-specific control
PARVA	alpha-parvin
PINCH-1	Particularly Interesting New Cysteine-Histidine rich protein
ROCK-1	Rho associated kinase-1
RSU-1	Ras suppressor-1
TGF-β	Transforming Growth Factor-β
