# Supplementary material for: Inhibition of Breast Cancer Cell Invasion by Ras Suppressor-1 (RSU-1) Silencing Is Reversed by Growth Differentiation Factor-15 (GDF-15)
Source: Int J Mol Sci. 2019 Jan 4;20(1):163. doi: 10.3390/ijms20010163 (PMC6337329; doi:10.3390/ijms20010163)
Supplement: Supplementary file 1 [file ijms-20-00163-s001.pdf]

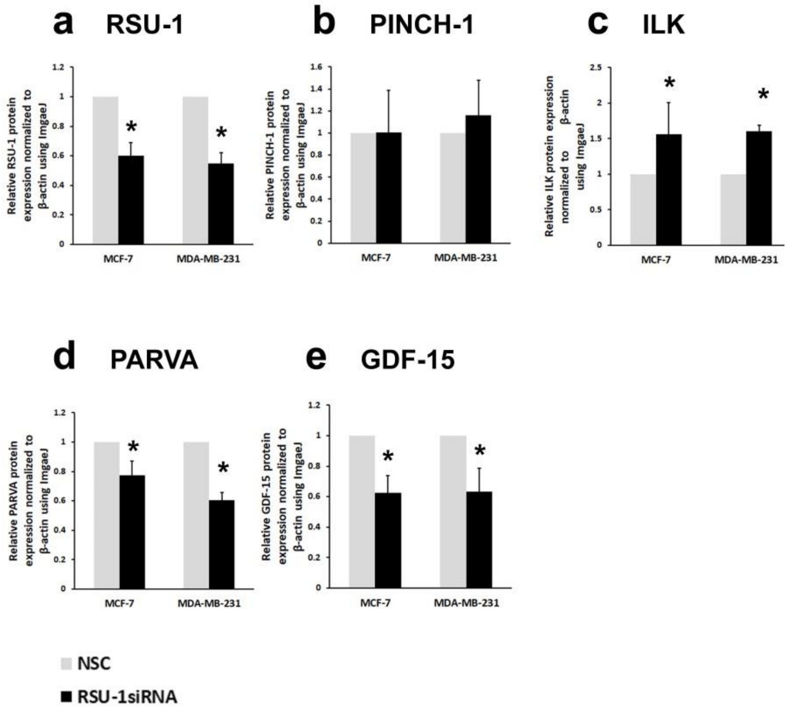

Supplementary Figure S1. Relative protein expression of *RSU-1, PINCH-1, ILK, PARVA* and *GDF-15* in MCF-7 and MDA-MB-231 cells following RSU-1 silencing. Protein expression quantification analysis of *RSU-1, PINCH-1, ILK, PARVA* and *GDF-15* was performed using the National Institute of Health Image J software. Several immunoblots were analyzed and the mean intensity of bands was normalized to that of β-actin which was used as loading control in each immunoblot. Relative protein expression of *RSU-1* (a) was quantified from seven (7) different immunoblots, relative protein expression of *PINCH-1* (b) was quantified from five (5) immunoblots, *ILK* (c) from two (2), *PARVA* (d) from three (3) and *GDF-15* (e) from four (4) immunoblots. Statistical significance corresponded to a p value of 0.05.

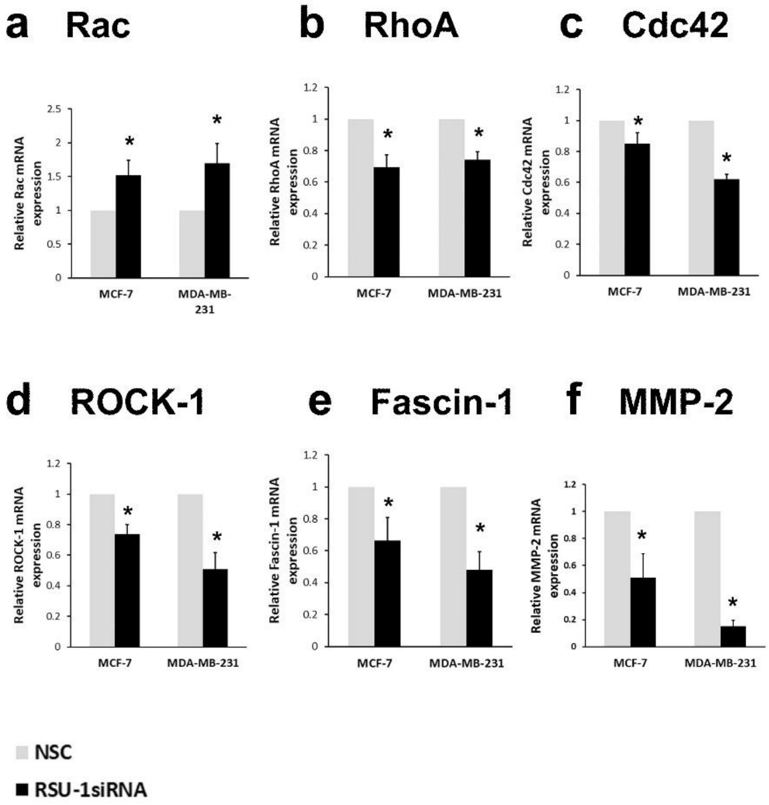

Supplementary Figure S2. The effect of *RSU-1* silencing on the mRNA expression of RhoGTPases, *ROCK-1*, *Fascin-1* and MMP2 in MCF-7 and MDA-MB-231 cells. Relative mRNA expression in MCF-7 and MDA-MB-231 cells of *Rac* (a), *RhoA* (b), *Cdc42* (c), *ROCK-2* (d), *Fascin-1* (e) and *MMP-2* (f) in cells transfected with NSC or RSU-1 siRNA. Three independent Real Time PCR experiments were performed, and data were analyzed using the ΔΔCt method and having the NSC-transfected cells as calibrators. Asterisks indicate statistically significant changes (p-value<0.05).

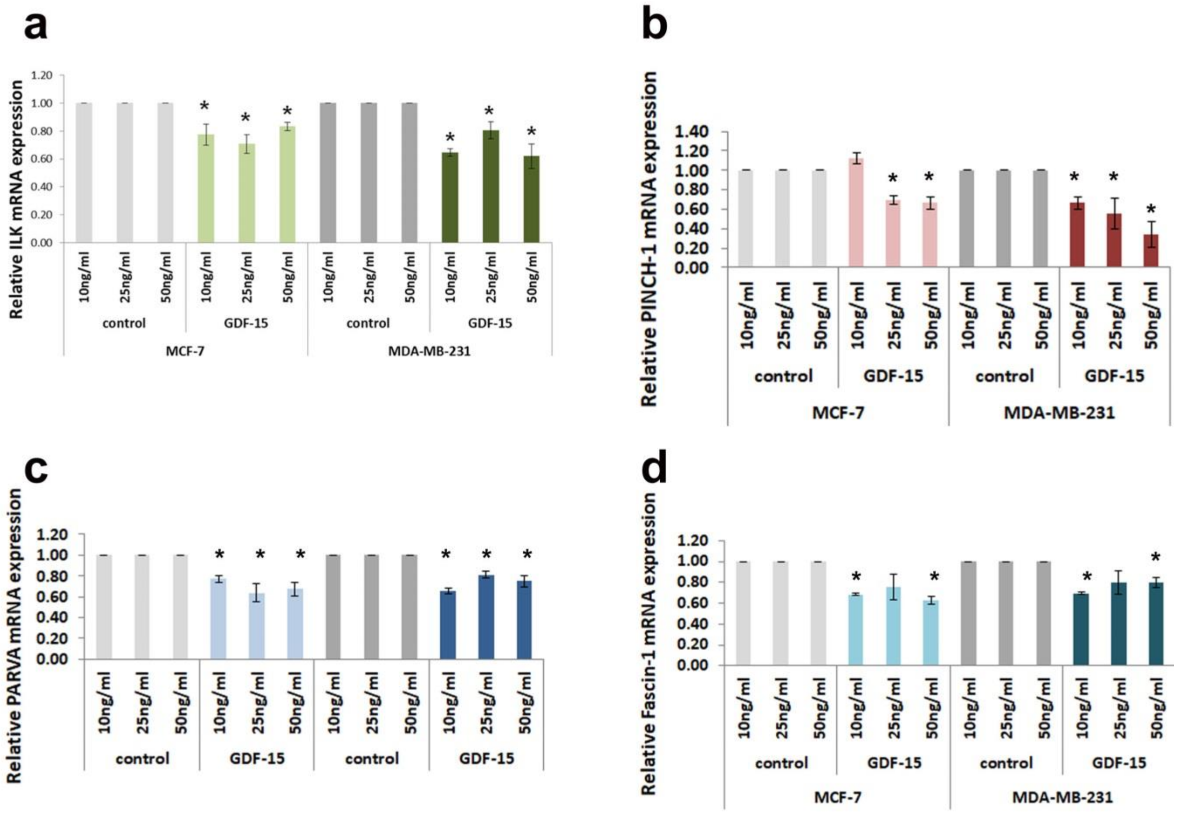

Supplementary Figure S3. Relative mRNA expression in MCF-7 and MDA-MB-231 cells of ILK *(a) PINCH-1 (b), PARVA (c) and Fascin-1 (d)* in cells treated with 10, 25 or 50ng/ml of GDF-15 for 24h. Two independent Real Time PCR experiments were performed, and data were analyzed using the ΔΔCt method and having the untreated sample as calibrator. The untreated sample per condition contained equal volume of solvent. Asterisks indicate statistically significant changes (p-value<0.05).

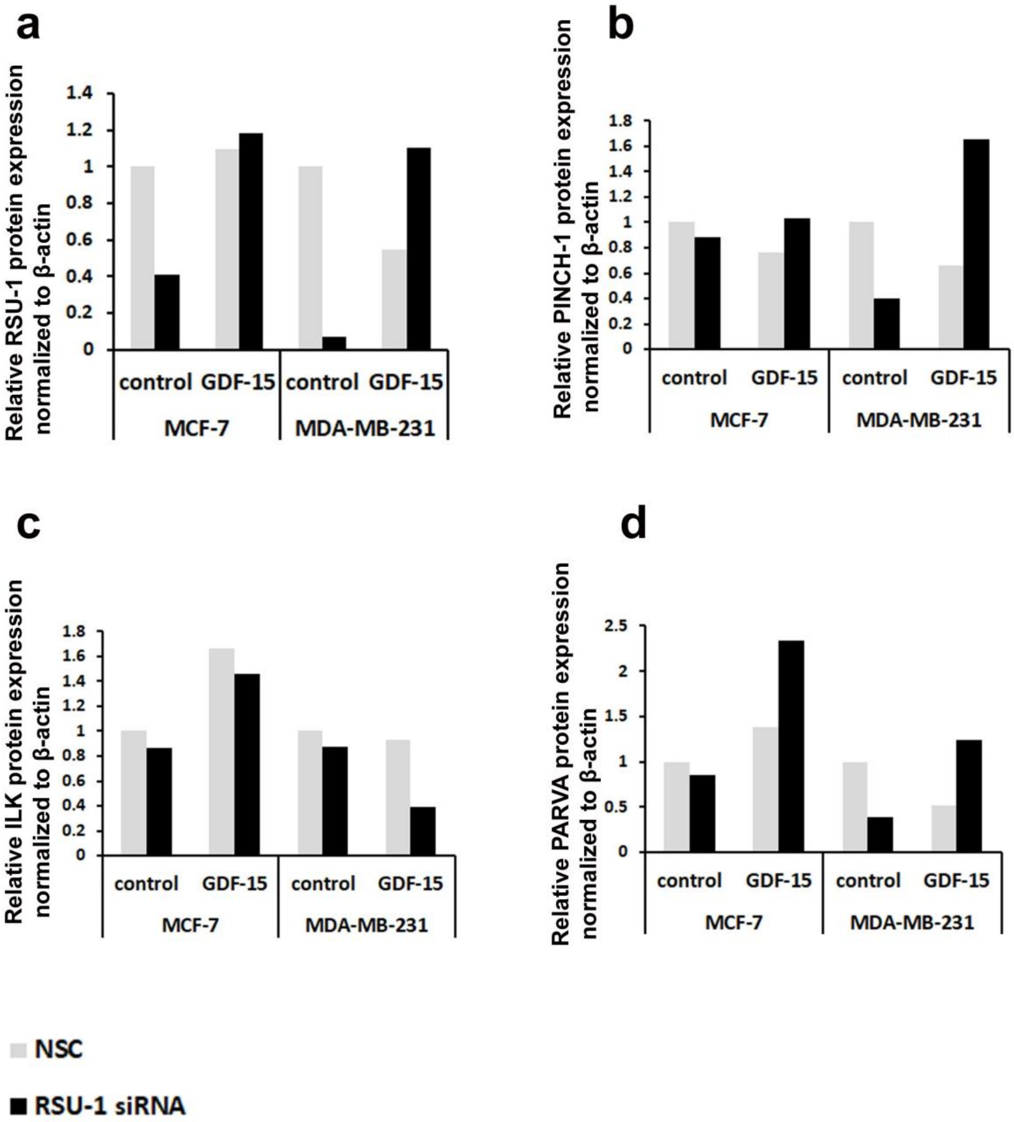

Supplementary Figure S4. Relative protein expression of *RSU-1*, *PINCH-1*, *ILK*, and *PARVA* in MCF-7 and MDA-MB-231 cells following RSU-1 silencing in the presence or absence of GDF-15. Protein expression quantification analysis was performed using the Image J software as described previously. The intensity of bands was normalized to that of β-actin and compared to the NSC-treated control (only solvent) sample in MCf-7 and MDA-MB-231 every time.
